# Knowledge, attitude, and practice of digital dentistry among dentists in rural and urban clinical settings in Western India: a descriptive cross-sectional study

**DOI:** 10.3389/froh.2026.1800833

**Published:** 2026-05-01

**Authors:** Sunanda Bhatnagar, Atrey J. Pai Khot, Amol Dhokar, Tanmay C. Juvekar, Vijay Bhavrao Desai

**Affiliations:** 1 Department of Oral Medicine and Radiology, T.P.C.T's Terna Dental College, Navi Mumbai, Maharashtra, India; 2 Department of Public Health Dentistry, Goa Dental College and Hospital, Bambolim-Goa, India; 3 Clinical Dental Sciences, College of Dentistry, Ajman University, Ajman, United Arab Emirates; 4 Center for Medical and Bio-Allied Health Sciences Research, Ajman University, Ajman, United Arab Emirates

**Keywords:** digital dentistry, healthcare disparities, rural, factors, urban

## Abstract

**Background:**

The adoption of digital technologies varies significantly across different regions, influenced by factors such as socioeconomic status, accessibility to resources, and educational opportunities. Maharashtra, one of the largest commercial states in India, with its diverse urban and rural settings, offers a unique opportunity to explore these variations among practicing dentists.

**Methods:**

A cross-sectional study design was implemented using a Google questionnaire distributed among 384 dentists from May to October 2024. Participants were selected using convenience sampling method and included dentists practicing across Maharashtra's urban centres and rural areas. Eligibility criteria included actively practicing dentists aged 25–65 years. Data was analysed using SPSS software (version 25, IBM Corporation, Armonk, NY, USA).00

**Results:**

The study identified a significant difference in knowledge and attitude scores between rural and urban dentists (*p* < 0.01), with urban practitioners displaying higher knowledge levels and more favourable attitudes towards digital dentistry. Practice scores were also higher in urban areas, though the correlation between knowledge and practice was weaker (*r* = 0.45), suggesting that additional factors like infrastructure and resources play a crucial role. Strong positive correlations were found between attitude and practice scores (*r* = 0.67, *p* < 0.001), emphasizing the importance of fostering positive attitudes for effective digital adoption.

**Conclusion:**

This study suggests disparities in the adoption of digital dentistry between rural and urban practice settings. These findings should be interpreted within the regional context and not generalized to all parts of India without further multi-state evidence. While urban practitioners benefit from better access to resources, rural dentists face challenges that limit the integration of digital tools into practice.

## Introduction

In the contemporary landscape of dentistry, technological advancements have catalysed a significant transformation, leading to the advent of digital dentistry which is a fusion of precision, innovation, and efficiency (1). This evolution marks the convergence of traditional dental practices with state-of-the-art digital tools and methodologies, revolutionizing the way dental care is delivered. Digital dentistry encompasses a broad spectrum of technological applications, ranging from digital imaging and CAD/CAM (Computer-Aided Design/ Computer-Aided Manufacturing) systems to 3D printing, digital impressions, and computer-guided surgeries (2). These innovations enhance diagnostic accuracy, treatment planning, patient experience, and overall clinical outcomes, presenting a paradigm shift in modern dental practices (3).

The origin of digital dentistry dates back to the 1980s, marked by Dr. François Duret's pioneering introduction of computer-aided design and computer-aided manufacturing (CAD/CAM) technology in dental applications, which laid the foundation for integrating computer technology into dental diagnostics and treatment (4). Over the years, the field has evolved from basic computer-assisted procedures to a fully integrated approach, with the introduction of intra-oral scanners, digital x-rays, and robotic-assisted surgeries amongst various advancements. The introduction of these tools has significantly reduced the need for physical impressions, minimized human error, and increased the speed and quality of dental procedures (5).

The integration of digital technologies into various dental disciplines has significantly influenced clinical workflows and treatment outcomes. In prosthodontics, advancements in computer-aided design and computer-aided manufacturing (CAD/CAM) have facilitated the fabrication of highly precise restorations, reducing material inconsistencies and improving marginal adaptation (6). Similarly, in Oral surgery, the utilization of three-dimensional imaging and computer-guided surgical planning has enhanced the accuracy of implant placement and complex surgical procedures, thereby minimizing intra-operative risks and post-operative complications (7). The CAD/CAM systems are also transforming the fields of endodontics and restorative dentistry, enhancing diagnostic precision and treatment efficiency (8). In endodontics, advancements such as cone-beam computed tomography (CBCT) and digital apex locators enable more accurate assessment of root canal morphology (9). Collectively, these digital advancements have led to greater efficiency, reproducibility, and precision across multiple dental specialties, ultimately contributing to improved patient care and clinical outcomes.

In India, the significance of digital dentistry cannot be overstated. The country's diverse geographical and demographic landscape presents unique challenges and opportunities for the integration of digital technologies in dental care. The growing urbanization, coupled with an increasing awareness of advanced dental procedures, has facilitated the adoption of digital tools in urban centres (10, 11). However, rural India, with its varied infrastructure and resource limitations, presents a different picture. Despite these challenges, the potential for digital dentistry to improve access to quality care in rural areas remains immense, given the ability to bridge the gaps in skill, infrastructure, and patient outcomes through tele-dentistry and remote consultations (12).

Maharashtra, one of India's most populous and economically diverse states, offers a unique setting for exploring the adoption and practice of digital dentistry. With its bustling urban centres such as Mumbai, Pune, and Nagpur stand at the forefront of technological advancements in healthcare. In contrast, the rural areas of the state, comprising numerous villages and small towns face distinct challenges in terms of healthcare infrastructure, access to education, and availability of advanced dental technologies. This juxtaposition of urban dynamism and rural resilience presents a fascinating backdrop to investigate how digital dentistry is perceived, adopted, and practiced across different settings (13).

This study aimed to explore the Knowledge, Attitudes and Practices (KAP) of dentists practicing in both rural and urban settings in Maharashtra, focusing on the varying levels of awareness, attitudes toward technological adoption, and the practical application of digital dentistry tools. This study sought to highlight the potential barriers and enables influencing the integration of digital technologies in dental practices across diverse geographical areas.

## Materials and methods

### Study design and setting

The study followed the design of a descriptive cross-sectional study conducted through an online questionnaire survey distributed to dentists practicing in both rural and urban areas of Maharashtra from May 2024 to October 2024.

### Ethical consideration and informed consent

The study was conducted adhering to the principles of the Declaration of Helsinki, after obtaining ethical clearance from the Institutional Ethics Committee (Approval number: TDC/EC/23/2024). An informed consent was obtained from all the participants before completing the questionnaire.

### Eligibility criteria and participants

#### Inclusion criteria

The study included the dentists practicing in Maharashtra. Practitioners from diverse settings, including private clinics, government hospitals, dental colleges, and community health centres. Dentists had to be aged between 25 and 65 years, and having a range of clinical experience and exposure to digital technologies.

#### Exclusion criteria

The participants above 65 years of age, not actively involved in clinical practice, such as researchers or educators were excluded from the study as they may have limited engagement with emerging digital technologies.

### Sample size estimation and sampling technique

The sample size was calculated using OpenEpi software (version 3.04) based on the results of a pilot study, which indicated a proportion of 81.5%. Z-score corresponding to a 95% confidence level. The following formula was used for sample size estimation:

$$n=Z2\times P(1-P)/e2n=Z^{2}\times P(1-P)/e^{2}n=Z2\times P(1-P)/e2$$

The sample size was calculated to be *n* = 384, accounting for 10% attrition, with Alpha (*α*) erro*r* = 0.05 and Power (1 − *β*) = 0.95. Participants were recruited through a convenience sampling method via professional dental networks and associations in Maharashtra. Although the minimum required sample size was estimated to be 384, the final analyzed sample comprised 250 complete responses (urban = 98; rural = 152), after exclusion of incomplete or ineligible responses.

### Questionnaire design and validation

The questionnaire was designed by the researchers based on the objectives of the study and essentially of 4 sections consisting of 39 close-ended questions. The first section encompassed the collection of demographic information followed by the further sections consisting of questions pertaining to knowledge, attitude and practices regarding digital dentistry, respectively. Detailed questionnaire items have now been provided as Supplementary File S1. The face and content validity of questionnaire were assessed by an expert committee consisting of eight subject matter experts for readability, clarity, and comprehensiveness of the questions. To evaluate the reliability of the questionnaire, Test-Retest reliability was measured using Kappa statistics. The resulting Cohen's kappa coefficient was determined to be 0.86, indicating a high level of agreement. Cronbach's alpha coefficient was calculated, yielding a value of 0.82, which demonstrates good internal consistency. The validity of the questionnaire was determined by means of face validity (0.84%) and content validity ratio (0.75).

### Grading system

The grading system employed was based on quartile derivatives. The knowledge, attitude and practice scores were computed by assigning one point for each accurate or positive response, and each incorrect or negative response received zero points. The final scores were given as a percentage after summing up each participant's points and calculating the percentages to assess the overall knowledge of the participants. The knowledge level was categorized as follows: High (>66%), Medium (33%–65%), and Low (<33%). Similarly, responses to attitude items were scored on a 5-point Likert scale as follows: 4 = strongly agree, 3 = agree, 2 = neutral, 1 = disagree, and 0 = strongly disagree. Moreover, practices regarding digital dentistry were graded as follows: 2: Routinely practices; 1: Sometimes; and 0: Never, following which cumulative scores were taken for evaluation of practices to categorize into Good practices (>50%) and Poor practices (<50%).

### Data collection and statistical analysis

The collected data were entered into Microsoft Excel (2009, Microsoft Corporation, Redmond, WA, USA) for initial organization and subsequently analyzed using SPSS software (version 25, IBM Corporation, Armonk, NY, USA) for statistical evaluation (14, 15). The statistical analysis involved descriptive as well as inferential methods. Descriptive statistics, including mean, standard deviation, and frequency distribution, were used to summarize continuous and categorical variables. Inferential statistics included the Chi-Square Test to compare categorical variables, such as rural versus urban responses, and the Independent T-Test to evaluate differences in mean knowledge and attitude scores between rural and urban dentists. Multiple Linear Regression Analysis was employed to identify predictors influencing the adoption of digital dentistry technologies. A significance level of *p* < 0.05 was considered for all analyses.

## Results

### Participants’ demographics

The demographic profile of the study participants revealed several key trends:

Age distribution showed that most dentists in both rural and urban settings were between 20 and 40 years. Gender distribution indicated a higher proportion of male dentists in rural areas (57.1%) compared to urban areas (48.7%), In terms of years of experience, a higher proportion of rural dentists had less than 5 years of experience (45.9%) whereas urban dentists had 5–10 years of experience (26.3%). Regarding qualifications, a larger percentage of rural dentists held a BDS degree (82.7%) compared to urban dentists had a MDS qualifications (42.8%). When it came to the type of practice, the majority of rural dentists were in private practice (89.8%) compared to 68.4% of urban dentists (Table 1).

**Table 1:** Demographic profile of study participants.

Demographic details	Urban *n* (%) [*n* = 98]	Rural *n* (%) [*n* = 152]
Age group (n%)		
20–30 years	54 (55.1)	90 (59.6)
31–40 years	30 (30.6)	43 (28.5)
41–50 years	13 (13.3)	15 (9.9)
>50 years	1 (1.0)	3 (2.0)
Gender (n%)		
Male	56 (57.1)	74 (48.7)
Female	42 (42.9)	78 (51.3)
Years of experience (n%)		
<5 years	45 (45.9)	73 (48.0)
5–10 years	35 (35.7)	40 (26.3)
10–15 years	10 (10.2)	27 (17.8)
>15 years	8 (8.2)	12 (7.9)
Qualification (n%)		
BDS	81 (82.7)	87 (57.2)
MDS	17 (17.3)	65 (42.8)
Type of practice (n%)		
Academic teaching hospital	1 (1.0)	21 (13.8)
Private practice	88 (89.8)	104 (68.4)
Both	9 (9.2)	27 (17.8)

All values are expressed as frequency and percentage (in parentheses).

### Comparison of knowledge, attitude and practices on digital dentistry among rural and urban dentists

**Figure 1:**
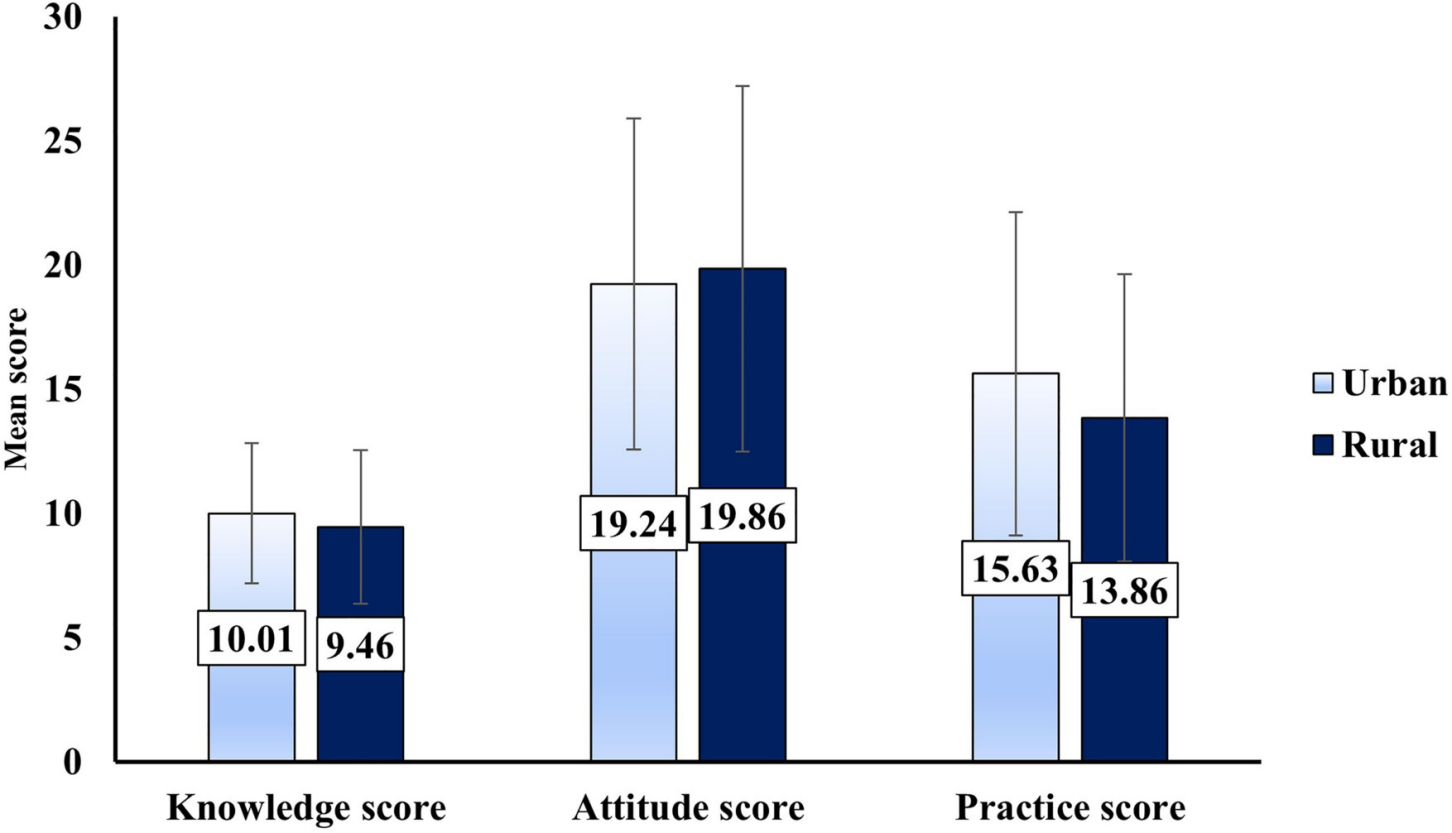
Comparison of knowledge, attitude and practice scores on digital dentistry among rural and urban dentists.

Urban dentists (86.7%) had a higher awareness of intra-oral cameras for routine examinations compared to urban dentists (77.0%), although the difference was not statistically significant (*P* = 0.140). Regarding optical scanners knowledge, there was a statistically significant difference (*P* = 0.05) between urban dentists (74.5%) compared to rural dentists (61.2%). For CAD/CAM applications, both rural (88.8%) and urban (85.5%) dentists showed similar knowledge, with no significant difference (*P* = 0.760). Awareness of AI applications in dentistry was also similar, with rural (55.1%) and urban (59.9%) dentists showing comparable levels of understanding, and no significant difference (*P* = 0.628) (Table 2). The attitudes toward digital dentistry showed similar perspectives across rural and urban dentists as depicted in Table 3. Both groups strongly agreed on the benefits of digital radiographs, though the difference was not statistically significant (*P* = 0.157). Similarly, a high percentage of both rural (60.2%) and urban (61.2%) dentists strongly agreed that intraoral scanners contribute to digital dentistry integration, with no significant difference (*P* = 0.643). Both groups also strongly agreed that 3D printing improves efficacy in dental procedures (rural: 52.0%, urban: 61.8%), with no significant difference (*P* = 0.248). Regarding CAD/CAM, most dentists in both groups agreed that it enhances precision and customization in treatment (rural: 54.1%, urban: 59.9%), with no significant difference (*P* = 0.345). However, a larger proportion of urban dentists (50.7%) strongly agreed that T-scan enhances occlusal analysis compared to rural dentists (37.8%), though this difference was not statistically significant (*P* = 0.067). A majority of both rural (82.7%) and urban (73.0%) dentists routinely used digital radiographs, and the difference was not statistically significant (*P* = 0.166). Urban dentists (52.0%) were more likely to routinely use intraoral cameras compared to rural dentists (30.9%), with a statistically significant difference (*P* = 0.008). The use of optical scanners was similar between urban (29.6%) and rural (22.4%) dentists, with no significant difference (*P* = 0.542). Both groups reported similar routine use of CAD/CAM, with rural dentists at 36.7% and urban dentists at 30.3%, but the difference was not statistically significant (*P* = 0.075) (Table 4). The comparison of knowledge, attitude, and practice scores between rural and urban dentists is depicted in Figure 1.

**Table 2:** Comparison of knowledge on digital dentistry among rural and urban dentists.

Knowledge questions	Urban (*n* = 98)	Rural (*n* = 152)	*χ* ^2^	*P*-value
Overall image quality of digital radiograph is superior compared to conventional radiograph				
Yes	89 (90.8%)	139 (91.4%)	0.113	0.945
No	8 (8.2%)	11 (7.2%)		
Don't know	1 (1.0%)	2 (1.3%)		
Awareness about the use of intraoral cameras for routine examinations				
Yes	85 (86.7%)	117 (77.0%)	3.925	0.140
No	11 (11.2%)	27 (17.8%)		
Don't know	2 (2.0%)	8 (5.3%)		
Dental procedures such as Aligners, Implant placement, Restorative dentistry and Prosthetics benefit the most from use of intraoral scanners.				
Yes	88 (89.8%)	124 (81.6%)	3.343	0.188
No	7 (7.1%)	17 (11.2%)		
Don't know	3 (3.1%)	11 (7.2%)		
Awareness of any of these optical scanners (Structured light scanner, Laser scanner, Confocal microscope scanner				
Yes	73 (74.5%)	93 (61.2%)	5.894	0.05*
No	16 (16.3%)	45 (29.6%)		
Don't know	9 (9.2%)	14 (9.2%)		
Familiarity with the DICOM(CBCT) and STL (format for 3D printing) integration				
Yes	76 (77.6%)	111 (73.0%)	0.945	0.623
No	16 (16.3%)	27 (17.8%)		
Don't know	6 (6.1%)	14 (9.2%)		
Awareness of CAD/CAM applications such as Crown and Bridge fabrication, Implant restoration, Orthodontic appliances and Denture design				
Yes	87 (88.8%)	130 (85.5%)	0.549	0.760
No	6 (6.1%)	12 (7.9%)		
Don't know	5 (5.1%)	10 (6.6%)		
Familiarity with T-scan in dentistry				
Yes	53 (54.1%)	85 (55.9%)	0.088	0.957
No	36 (36.7%)	54 (35.5%)		
Don't know	9 (9.2%)	13 (8.6%)		
Accuracy of digital shade matching is superior compared to conventional methods				
Yes	71 (72.4%)	103 (67.8%)	2.829	0.243
No	14 (14.3%)	34 (22.4%)		
Don't know	13 (13.3%)	15 (9.9%)		
Digital face scanner allows collaboration between dental specialists				
Yes	71 (72.4%)	102 (67.1%)	0.798	0.671
No	14 (14.3%)	26 (17.1%)		
Don't know	13 (13.3%)	24 (15.8%)		
Improvement in the aesthetics of smile with digital smile designing				
Yes	86 (87.8%)	125 (82.2%)	2.041	0.360
No	8 (8.2%)	14 (9.2%)		
Don't know	4 (4.1%)	13 (8.6%)		
Preference for guided implant technology over conventional implant placement methods				
Yes	77 (78.6%)	111 (73.0%)	1.912	0.384
No	15 (15.3%)	24 (15.8%)		
Don't know	6 (6.1%)	17 (11.2%)		
Tele dentistry enables access to larger population for dental care				
Yes	71 (72.4%)	107 (70.4%)	0.181	0.914
No	13 (13.3%)	23 (15.1%)		
Don't know	14 (14.3%)	22 (14.5%)		
Familiarity with any one of the applications of artificial intelligence (AI) in Dentistry: Low Dose Metal Artefact Reduction, (MAR) in CBCT, CBCT airway analysis, Navi dent, Maestro 3D ortho and VGG 16, VGG 19				
Yes	54 (55.1%)	91 (59.9%)	0.930	0.628
No	32 (32.7%)	41 (27.0%)		
Don't know	12 (12.2%)	20 (13.2%)		
Impact of integrating digital technology in your dental practice				
Reduced treatment time	20 (20.4%)	24 (15.8%)	4.574	0.470
Improved diagnostic accuracy	24 (24.5%)	44 (28.9%)		
Streamlined workflow efficiency	3 (3.1%)	8 (5.3%)		
Improved overall satisfaction	21 (21.4%)	34 (22.4%)		
Enhanced patient communication	25 (25.5%)	28 (18.4%)		
Enhanced treatment planning	5 (5.1%)	14 (9.2%)		

All values are expressed as frequency and percentage (in parentheses). Statistical test used: Chi square test. Level of significance:

^*^ *P*-value ≤ 0.05 is considered statistically significant.

**Table 3:** Comparison of attitude on digital dentistry among rural and urban dentists.

Attitude questions	Urban (*n* = 98)	Rural (*n* = 152)	χ^2^	*P*-value
Using digital radiograph such as RVG and PSP technology benefits in terms of higher resolution, quicker image retriever and decrease radiation exposure				
Strongly disagree	0 (0.0%)	0 (0.0%)	5.204	0.157
Disagree	0 (0.0%)	2 (1.3%)		
Neutral	1 (1.0%)	5 (3.3%)		
Agree	28 (28.6%)	29 (19.1%)		
Strongly agree	69 (70.4%)	116 (76.3%)		
The additional features of intraoral cameras such as improved resolution, enhanced ergonomic design, with AI for diagnostics is for better integration				
Strongly disagree	0 (0.0%)	0 (0.0%)	3.596	0.309
Disagree	1 (1.0%)	3 (2.0%)		
Neutral	11 (11.2%)	8 (5.3%)		
Agree	38 (38.8%)	57 (37.5%)		
Strongly agree	48 (49.0%)	84 (55.3%)		
Intraoral scanners contribute to digital dentistry integration by seamless connection with CAD/CAM systems, real time collaboration with dental laboratories and enhancing treatment documentation				
Strongly disagree	0 (0.0%)	0 (0.0%)	1.675	0.643
Disagree	1 (1.0%)	1 (0.7%)		
Neutral	8 (8.2%)	19 (12.5%)		
Agree	30 (30.6%)	39 (25.7%)		
Strongly agree	59 (60.2%)	93 (61.2%)		
Crucial aspects in selecting an optical scanner are speed of scanning, accuracy of scans, ease of use, cost and compatibility with CAD/CAM systems				
Strongly disagree	0 (0.0%)	0 (0.0%)	2.186	0.535
Disagree	0 (0.0%)	2 (1.3%)		
Neutral	13 (13.3%)	21 (13.8%)		
Agree	37 (37.8%)	48 (31.6%)		
Strongly agree	48 (49.0%)	81 (53.3%)		
3D printing technology has improved efficacy of crown and bridge fabrication, implant drill guide, maxillofacial prosthetics, occlusal splints and in regenerative dentistry.				
Strongly disagree	0 (0.0%)	0 (0.0%)	4.128	0.248
Disagree	1 (1.0%)	2 (1.3%)		
Neutral	9 (9.2%)	17 (11.2%)		
Agree	37 (37.8%)	39 (25.7%)		
Strongly agree	51 (52.0%)	94 (61.8%)		
CAD/CAM technology enhances the precision, faster treatment, improved aesthetics and patient customisation				
Strongly disagree	0 (0.0%)	0 (0.0%)	3.323	0.345
Disagree	0 (0.0%)	3 (2.0%)		
Neutral	10 (10.2%)	11 (7.2%)		
Agree	35 (35.7%)	47 (30.9%)		
Strongly agree	53 (54.1%)	91 (59.9%)		
T scan technology enhances occlusal analysis and contributes to longevity of dental restorations				
Strongly disagree	0 (0.0%)	0 (0.0%)	7.165	0.067
Disagree	2 (2.0%)	3 (2.0%)		
Neutral	23 (23.5%)	39 (25.7%)		
Agree	36 (36.7%)	33 (21.7%)		
Strongly agree	37 (37.8%)	77 (50.7%)		
The digital shade guide has streamlined the process of selecting the appropriate tooth colour for restoration hence saving time and increasing accuracy				
Strongly disagree	1 (1.0%)	0 (0.0%)	4.491	0.344
Disagree	2 (2.0%)	1 (0.7%)		
Neutral	12 (12.2%)	24 (15.8%)		
Agree	43 (43.9%)	55 (36.2%)		
Strongly agree	40 (40.8%)	72 (47.4%)		
Digital face scanner helps in achieving better aesthetic outcomes in dental procedures.				
Strongly disagree	1 (1.0%)	1 (0.7%)	5.588	0.232
Disagree	2 (2.0%)	1 (0.7%)		
Neutral	12 (12.2%)	26 (17.1%)		
Agree	41 (41.8%)	45 (29.6%)		
Strongly agree	42 (42.9%)	79 (52.0%)		
Incorporating digital smile design in dentistry benefits in terms of faster treatment planning, more accurate outcomes, enhanced patient communication and improved aesthetics				
Strongly disagree	0 (0.0%)	0 (0.0%)	2.975	0.396
Disagree	1 (1.0%)	4 (2.6%)		
Neutral	6 (6.1%)	17 (11.2%)		
Agree	37 (37.8%)	49 (32.2%)		
Strongly agree	54 (55.1%)	82 (53.9%)		
In dental practice, Computer Guided Implant technology benefits in terms of improved accuracy in implant placement, enhanced treatment planning, reduced surgery time and better patient outcome				
Strongly disagree	0 (0.0%)	0 (0.0%)	3.283	0.350
Disagree	0 (0.0%)	1 (0.7%)		
Neutral	10 (10.2%)	19 (12.5%)		
Agree	41 (41.8%)	48 (31.6%)		
Strongly agree	47 (48.0%)	84 (55.3%)		
Tele dentistry services are the most beneficial in remote consultations, virtual follow-ups, telediagnosis and digital marketing of dental treatment.				
Strongly disagree	0 (0.0%)	1 (0.7%)	1.225	0.874
Disagree	1 (1.0%)	2 (1.3%)		
Neutral	19 (19.4%)	24 (15.8%)		
Agree	32 (32.7%)	53 (34.9%)		
Strongly agree	46 (46.9%)	72 (47.4%)		
AI tools have the potential to streamline administrative tasks in dental offices.				
Strongly disagree	1 (1.0%)	1 (0.7%)	5.403	0.248
Disagree	3 (3.1%)	0 (0.0%)		
Neutral	18 (18.4%)	25 (16.4%)		
Agree	32 (32.7%)	48 (31.6%)		
Strongly agree	44 (44.9%)	78 (51.3%)		
The challenges encountered in the process of implementing digital technology in your dental practice are initial cost of equipment, staff training difficulties or the integration issues with existing systems.				
Strongly disagree	0 (0.0%)	1 (0.7%)	2.258	0.688
Disagree	1 (1.0%)	0 (0.0%)		
Neutral	9 (9.2%)	15 (9.9%)		
Agree	36 (36.7%)	54 (35.5%)		
Strongly agree	52 (53.1%)	82 (53.9%)		

All values are expressed as frequency and percentage (in parentheses). Statistical test used: Chi square test. Level of significance:

^*^ *P*-value ≤ 0.05 is considered statistically significant.

**Table 4:** Comparison of practice on digital dentistry among rural and urban dentists.

Practice questions	Urban (*n* = 98)	Rural (*n* = 152)	χ^2^	*P*-value
Use of digital radiograph in your clinic/hospital practice				
Routinely	81 (82.7%)	111 (73.0%)	5.084	0.166
Sometimes	14 (14.3%)	33 (21.7%)		
Never	3 (3.1%)	4 (2.6%)		
Not applicable	0 (0.0%)	4 (2.6%)		
Use of intraoral cameras in diagnosing dental conditions				
Routinely	51 (52.0%)	47 (30.9%)	11.950	0.008*
Sometimes	34 (34.7%)	78 (51.3%)		
Never	12 (12.2%)	22 (14.5%)		
Not applicable	1 (1.0%)	5 (3.3%)		
Difficulties with patient co-operation while using intraoral scanner				
Routinely	27 (27.6%)	34 (22.4%)	1.221	0.748
Sometimes	47 (48.0%)	73 (48.0%)		
Never	16 (16.3%)	30 (19.7%)		
Not applicable	8 (8.2%)	15 (9.9%)		
Use of optical scanner in your dental practice				
Routinely	29 (29.6%)	34 (22.4%)	2.147	0.542
Sometimes	42 (42.9%)	66 (43.4%)		
Never	19 (19.4%)	35 (23.0%)		
Not applicable	8 (8.2%)	17 (11.2%)		
Challenges while integration of DICOM and STL files				
Routinely	34 (34.7%)	37 (24.3%)	4.924	0.177
Sometimes	39 (39.8%)	62 (40.8%)		
Never	11 (11.2%)	30 (19.7%)		
Not applicable	14 (14.3%)	23 (15.1%)		
Limitations due to cost and technical complexities in your use of CAD/CAM				
Routinely	36 (36.7%)	46 (30.3%)	6.901	0.075
Sometimes	39 (39.8%)	84 (55.3%)		
Never	14 (14.3%)	11 (7.2%)		
Not applicable	9 (9.2%)	11 (7.2%)		
Use of T-scan to correct occlusal discrepancies to help enhance patient outcome				
Routinely	30 (30.6%)	35 (23.0%)	3.493	0.322
Sometimes	32 (32.7%)	61 (40.1%)		
Never	26 (26.5%)	34 (22.4%)		
Not applicable	10 (10.2%)	22 (14.5%)		
Use of digital shade guide in your dental practice				
Routinely	33 (33.7%)	43 (28.3%)	2.034	0.565
Sometimes	38 (38.8%)	65 (42.8%)		
Never	20 (20.4%)	27 (17.8%)		
Not applicable	7 (7.1%)	17 (11.2%)		
Use of digital face scanner for aesthetics, evaluating lip dynamics, assessing craniofacial growth or capturing tooth morphology				
Routinely	39 (39.8%)	41 (27.0%)	7.849	0.049*
Sometimes	32 (32.7%)	51 (33.6%)		
Never	21 (21.4%)	36 (23.7%)		
Not applicable	6 (6.1%)	24 (15.8%)		
Limitations due to cost, learning curve and technical issues in your use of computer guided implant technology				
Routinely	40 (40.8%)	48 (31.6%)	6.494	0.090
Sometimes	36 (36.7%)	74 (48.7%)		
Never	18 (18.4%)	18 (11.8%)		
Not applicable	4 (4.1%)	12 (7.9%)		
Benefits by use of tele dentistry for remote consultations, virtual follow-ups and treatment planning discussions				
Routinely	46 (46.9%)	39 (25.7%)	12.116	0.007*
Sometimes	32 (32.7%)	70 (46.1%)		
Never	12 (12.2%)	24 (15.8%)		
Not applicable	8 (8.2%)	19 (12.5%)		
Use of AI tools in diagnosis and treatment planning				
Routinely	42 (42.9%)	41 (27.0%)	8.142	0.043*
Sometimes	29 (29.6%)	65 (42.8%)		
Never	18 (18.4%)	26 (17.1%)		
Not applicable	9 (9.2%)	20 (13.2%)		
Recommendation for addressing the learning curve associated with adopting new digital technologies				
Routinely	56 (57.1%)	79 (52.0%)	3.380	0.337
Sometimes	30 (30.6%)	55 (36.2%)		
Never	11 (11.2%)	12 (7.9%)		
Not applicable	1 (1.0%)	6 (3.9%)		

All values are expressed as frequency and percentage (in parentheses). Statistical test used: Chi square test. Level of significance:

^*^ *P*-value ≤ 0.05 is considered statistically significant.

### Correlation and association between knowledge, attitude and practices scores of the study participants

A positive linear correlation and a high statistically significant difference (*P* < 0.001) was found between the knowledge, attitude and practice scores among rural and urban dentists by the Spearman's rank correlation coefficient test (Table 5). Multiple linear regression analysis model showed a statistically significant relationship between knowledge score with age (95%CI: −1.74–(−0.25); *p* = 0.009), and location (95%CI: −1.58–(−0.01); *p* = 0.047). The attitude score of the participants showed a similar relationship with gender (95%CI: −4.91–(−1.22); *P* = 0.001), whereas practice score showed significant association with location (95%CI: −3.48–0.23; *P* = 0.025). The field-wise data are presented in (Table 6).

**Table 5:** Correlation between knowledge score with attitude and practice scores of the study participants.

Scores		Attitude	Practice
Knowledge	Spearman correlation coefficient	0.543	0.444
	*P*-value	<0.001*	<0.001*

Statistical test used: Spearman correlation test. Level of significance:

^*^ *P*-value ≤ 0.05 is considered statistically significant.

**Table 6:** Association between demographic variables and knowledge/attitude/practice scores of the study participants.

Predictors	Coefficient r	SE	t	95% CI	*P*-value	Adjusted R2
Dependent variable: Knowledge score						
(Constant)	12.070	1.345	8.973	9.420–14.719	<0.001*	0.049
Age	−0.996	0.377	−2.644	−1.738 to −0.254	0.009*	
Gender	−0.454	0.395	−1.148	−1.232 to 0.325	0.252	
Location	−0.796	0.399	−1.996	−1.582 to −0.010	0.047*	
Years of experience	−0.073	0.293	−0.248	−0.650 to 0.505	0.804	
Qualification	0.854	0.443	1.929	−0.018 to 1.726	0.055	
Type of Practice	0.053	0.411	0.129	−0.756 to 0.862	0.897	
Dependent variable: Attitude score						
(Constant)	25.634	3.192	8.031	19.347 to 31.922	<0.001*	0.046
Age	−1.448	0.894	−1.620	−3.209 to 0.313	0.107	
Gender	−3.067	0.938	−3.269	−4.915 to −1.219	0.001*	
Location	0.217	0.947	0.229	−1.648 to 2.082	0.819	
Years of experience	−0.501	0.696	−0.721	−1.872 to 0.869	0.472	
Qualification	1.999	1.051	1.903	−0.070 to 4.069	0.058	
Type of practice	−0.647	0.974	−0.663	−2.566 to 1.273	0.508	
Dependent variable: Practice score						
(Constant)	17.001	2.778	6.119	11.528 to 22.474	<0.001*	0.030
Age	−0.804	0.778	−1.033	−2.337 to 0.729	0.303	
Gender	0.197	0.817	0.242	−1.411 to 1.806	0.809	
Location	−1.856	0.824	−2.252	−3.479 to −0.232	0.025*	
Years of experience	−0.525	0.606	−0.867	−1.718 to 0.668	0.387	
Qualification	0.105	0.915	0.115	−1.697 to 1.907	0.909	
Type of practice	1.114	0.848	1.314	−0.557 to 2.785	0.190	

CI, Confidence interval; SE, standard error. Statistical test used: Multiple linear regression analysis. Level of significance:

^*^ *P* value ≤ 0.05 is considered statistically significant.

## Discussion

The significance of understanding the knowledge, attitudes, and practices (KAP) regarding digital dentistry is amplified in today's era. In urban areas, the adoption of digital technologies is often driven by access to resources, advanced training, and a more progressive approach to healthcare. However, in rural regions, factors such as limited access to education, financial constraints, and infrastructural limitations can impede the widespread adoption of these technologies (16). This disparity underscores the need for a detailed exploration of how digital dentistry is understood and implemented by dental practitioners in different parts of Maharashtra.

The findings of this study provide valuable insights into the knowledge, attitudes, and practices of rural and urban dentists regarding digital dentistry, offering implications for education, policy-making, and resource allocation in dental practices. The results reveal that both rural and urban dentists possess a relatively high level of knowledge about digital dentistry, with regional variations reflecting differing levels of exposure to specific technologies. While urban dentists demonstrated greater awareness of optical scanners, likely due to their lower cost and accessibility, both groups exhibited a strong understanding of digital radiographs, CAD/CAM systems, and AI applications in dentistry. These findings align with those of Inampudi et al. (16) and Emami et al. (17) who highlighted global trends emphasizing the integration of digital technologies into dental education through academic curricula and continuing education programs (16, 17).

Attitudes toward digital dentistry were overwhelmingly positive among participants, with widespread agreement on the benefits of digital radiographs, intraoral scanners, and CAD/CAM systems in improving diagnostic accuracy, treatment outcomes, and patient satisfaction. However, disparities emerged in attitudes toward more advanced technologies such as 3D printing and T-scan systems. Rural dentists expressed lower enthusiasm for adopting these tools, likely due to limited exposure and access to advanced technologies in their practice settings. In contrast, urban dentists appeared more inclined to embrace these innovations, benefiting from better access to cutting-edge equipment and diverse professional environments, including academic and hospital-based settings. These differences underline the importance of fostering equitable access to advanced digital tools and addressing geographic disparities in technological adoption.

The practical application of digital dentistry tools highlighted further distinctions between rural and urban dentists. Emami et al. (17) and Pentapati et al. (18) reported higher usage of intraoral cameras, a cost-effective tool that enhances diagnostic communication and is more accessible in resource-limited settings such as those of rural practitioners (17, 18). Conversely, urban dentists showed greater adoption of digital radiographs, facilitated by superior infrastructure and availability of resources. The use of technologies such as optical scanners, CAD/CAM systems, and T-scan devices was more evenly distributed, reflecting a general recognition of their utility (2). However, the implementation of these tools is often influenced by external factors such as financial constraints, infrastructure, and practice type. These findings suggest that while both rural and urban dentists acknowledge the importance of digital tools, their practical adoption remains contingent upon overcoming logistical and economic barriers. Dentists with greater knowledge of digital tools tended to exhibit more positive attitudes toward their adoption, which in turn increased the likelihood of integrating these tools into clinical practice. However, the moderate correlation between knowledge and practice (*r* = 0.45) underscores the influence of external factors such as infrastructure, cost, and institutional support. This highlights the need for targeted interventions that not only enhance knowledge and attitudes but also address practical barriers to adoption, particularly in rural areas.

Regional differences in digital dentistry adoption were a prominent finding. Urban dentists exhibited greater engagement with accessible tools such as intraoral cameras, reflecting their practicality and affordability in resource-constrained settings. Urban dentists benefited from better infrastructure and access to advanced technologies, facilitating broader adoption of digital tools. These disparities underscore the necessity of ensuring equitable access to digital technologies across geographic locations to mitigate the risk of unequal quality of care. Policy makers and dental organizations should prioritize strategies that bridge this digital divide, such as subsidies, funding initiatives, and collaborative partnerships between rural and urban practices. This study's findings align with previous research while offering novel insights into the barriers and opportunities associated with digital dentistry in rural and urban settings. Younger dentists and those with advanced qualifications, such as MDS, were predominantly concentrated in urban areas, consistent with studies by Vasupriya et al. (19). This trend can be attributed to the availability of advanced training facilities and better career prospects in urban regions.

Awareness and adoption rates of digital tools such as intraoral scanners and CAD/CAM systems were significantly higher among urban dentists, reflecting trends observed by Nayakar et al. (10). Urban practitioners often benefit from workshops, conferences, and online resources that promote digital dentistry, whereas rural dentists face limited access to such opportunities (20). Low awareness of emerging technologies like 3D printing among both groups, with slightly higher rates in urban areas, mirrors the observations of Acharya et al. (21), who highlighted the nascent stage of 3D printing adoption in global dental practices. High costs and limited training were identified as primary barriers to adoption echoing findings by Flores-Mir et al. (22) and Zande et al. (23).

The resistance to adopting digital tools due to financial constraints and lack of training was prevalent, especially in rural areas (24). High initial investments required for advanced tools such as intraoral scanners and CAD/CAM systems pose significant challenges for rural dentists, who often operate in low-revenue practices (25). The lack of targeted training programs tailored for rural practitioners exacerbates this issue, as noted by Zande et al. (23). Resistance to change, driven by fear of failure and comfort with traditional methods, was equally observed in both groups, consistent with findings by Suganna et al. (26). Addressing these barriers requires a multifaceted approach, including financial incentives, hands-on training workshops, and motivational success stories. Urban dentists reported higher perceived benefits of digital tools in terms of time efficiency and patient satisfaction, aligning with findings by Khurshid et al. (27). Urban practices, characterized by higher patient inflow and demand for advanced services, are more likely to justify the return on investment in digital tools. Awareness campaigns emphasizing the long-term advantages of digital tools could help bridge this perception gap and motivate rural dentists to adopt these technologies (28, 29).

The DigiDent-KAPAT can serve as an invaluable monitoring and evaluation instrument, enabling stakeholders to measure the impact of training initiatives over time. The global comparison underscores the digital divide between high-income and low- to middle-income countries. High-income countries have a significantly higher adoption rates of digital technologies across rural and urban settings, supported by better funding and government incentives. The stark contrast observed in this study highlights the urgent need for policy interventions to enhance access to digital tools in countries like India.

A limitation of this study is its geographical scope, as it focuses only on Western, Peninsular Part of India, which may not fully represent digital dentistry adoption across India. It relies on self-reported data which introduces potential recall and social desirability biases, affecting result accuracy, and long-term adoption trends. Furthermore, the exclusion of key stakeholders, such as policymakers and industry experts, restricts a comprehensive understanding of the barriers and opportunities in digital dentistry integration. In addition to the restricted geographic scope, the use of convenience sampling through professional dental networks may have introduced selection bias, potentially overrepresenting dentists who are more digitally active, professionally connected, or inclined to respond to online surveys. Therefore, the findings should be interpreted cautiously and not considered representative of all dentists in Maharashtra or India. Future studies should employ stratified or probability-based sampling across districts and practice settings to improve representativeness.

Future research should expand to multiple states and adopt a longitudinal approach to track evolving trends. Interventional studies evaluating the impact of hands-on training, financial subsidies, and online programs can help bridge urban-rural gaps in technology adoption. Cost-benefit analyses of digital tools in different practice settings could guide policy decisions on funding and infrastructure support. Comparative studies with other developing nations may also provide strategies to overcome common adoption barriers. Finally, integrating AI-driven insights into digital dentistry research and advocating for policy measures, such as subsidies and mandatory training, can facilitate wider and more equitable adoption, ultimately improving patient care and professional practice.

## Conclusion

While both the groups demonstrated a strong understanding of digital tools, urban dentists exhibited higher adoption rates, particularly for advanced technologies like CAD/CAM and 3D printing, due to better infrastructure and training opportunities. Urban dentists relied more on cost-effective tools like intraoral cameras and optical scanners. The findings underscore the need for targeted interventions to bridge this digital divide, ensuring equitable access to advanced dental technologies. Addressing these disparities through financial incentives, hands-on training, and policy support will be crucial for enhancing digital dentistry adoption across diverse practice settings.

## Data Availability

The raw data supporting the conclusions of this article will be made available by the authors, without undue reservation.
