# Supplementary material for: Knowledge, attitude, and practice of digital dentistry among dentists in rural and urban clinical settings in Western India: a descriptive cross-sectional study
Source: Front Oral Health. 2026 May 1;7:1800833. doi: 10.3389/froh.2026.1800833 (PMC13176248; doi:10.3389/froh.2026.1800833)
Supplement: Supplementary file 1 [file Datasheet1.pdf]

Digital Dentistry: Knowledge, Attitude and Practice amongst Dentists in Rural Vs Urban Settings in the State of Maharashtra - A Questionnaire based Study

Questionnaire

Knowledge/awareness-based question

Sr No.	Questions	Yes	No	Don't know
1	Overall image quality of digital radiograph is superior compared to conventional radiograph			
2	Are you aware about the use of intraoral cameras for routine examinations?			
3	Dental procedures such as Aligners, Implant placement, Restorative dentistry and Prosthetics benefits the most from use of intraoral scanners.			
4	Are you aware of any of the optical scanners(Structured light scanner, Laser scanner, Confocal microscope scanner)?			
5	Are you familiar with the DICOM(CBCT) and STL(format for 3D printing) integration?			
6	Are you aware of CAD/CAM applications such as Crown and Bridge fabrication, Implant restoration, Orthodontic appliances and Denture design?			
7	Are you familiar with T-scan technology in dentistry?			
8	Do you think the accuracy of digital shade matching is superior compared to conventional methods?			
9	Digital face scanner allows collaboration between dental specialists.			
10	Do you think digital smile designing can improve the aesthetics of a smile?			

11	Do you think computer-guided implant technology is preferable over conventional implant placement methods?			
12	Teledentistry enables access to larger population for dental care.			
13	Are you familiar with any one of the applications of artificial intelligence (AI) in Dentistry : Low Dose Metal Artefact Reduction,(MAR) in CBCT, CBCT airway analysis, Navi dent, Maestro 3D ortho and VGG 16, VGG 19?			

Attitude based questions

Sr No	Questions	Strongly agree	Agree	Neutral	Disagree	Strongly disagree
1	Using digital radiograph such as RVG and PSP technology benefits in terms of higher resolution, quicker image retriever and decrease radiation exposure.					
2	The additional features of intraoral cameras such as improved resolution, enhanced ergonomic design, with AI for diagnostics is for better integration.					
3	Intraoral scanners contribute to digital dentistry integration by seamless connection with CAD/CAM systems, real time collaboration with dental laboratories and enhancing treatment documentation.					
4	Crucial aspect in selecting an optical scanner are speed of scanning, accuracy of scans, ease of use, cost and compatibility with CAD/CAM systems.					

5	3D printing technology has emproved efficacy of crown and bridge fabrication, implant drill guide,maxillofacial prosthetics, occlusal splints and in regenerative dentistry.					
6	CAD/CAM technology enhances the precision ,faster treatment, improved aesthetics and patient customisation.					
7	T scan technology enhances occlusal analysis and contributes to longevity of dental restorations.					
8	The digital shade guide has streamlined the process of selecting the appropriate tooth colour for restoration hence saving time and increasing accuracy.					
9	Digital face scanner helps in achieving better aesthetic outcomes in dental procedures.					
10	Incorporating digital smile design in dentistry benefits in terms of faster treatment planning, more accurate outcomes, enhanced patient communication and improved aesthetics.					
11	In dental practice, Computer Guided Implant technology benefits in terms of improved accuracy in implant placement, enhanced treatment planning, reduced surgery time and better patient outcome.					
12	AI tools have the potential to streamline administrative tasks in dental offices.					

13	The challenges encountered in the process of implementing digital technology in your dental practice are initial cost of equipment, staff training difficulties or the integration issues with existing systems.					

Practice based question

Sr. No	Questions	Routinely	Sometimes	Never	Not applicable
1	Use of digital radiograph in your clinic/hospital practice.				
2	Do you use intraoral cameras in diagnosing dental conditions?				
3	Do you encounter difficulties with patient co-operation while using intraoral scanner?				
4	Use of optical scanner in your dental practice.				
5	Do you encounter challenges while integration of DICOM and STL files in your digital dental practice?				
6	Does cost and technical complexities limit your use of CAD/CAM in dental practice?				
7	Do you use T-scan to correct occlusal discrepancies to help enhance patient outcome.				
8	Use of digital shade guide in your dental practice.				
9	Do you use digital face scanner for aesthetics, evaluating lip dynamics, assessing craniofacial growth or capturing tooth morphology in your dental practice?				
10	Does cost, learning curve and technical issues limit your use of computer guided implant technology in dental practice?				

11	Are you benefited by use of teledentistry for remote consultations, virtual follow-ups and treatment planning discussions in your practice?				
12	Use of AI tools in diagnosis and treatment planning.				
13	Would you recommend addressing the learning curve associated with adopting new digital technologies in dental practice?				
